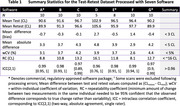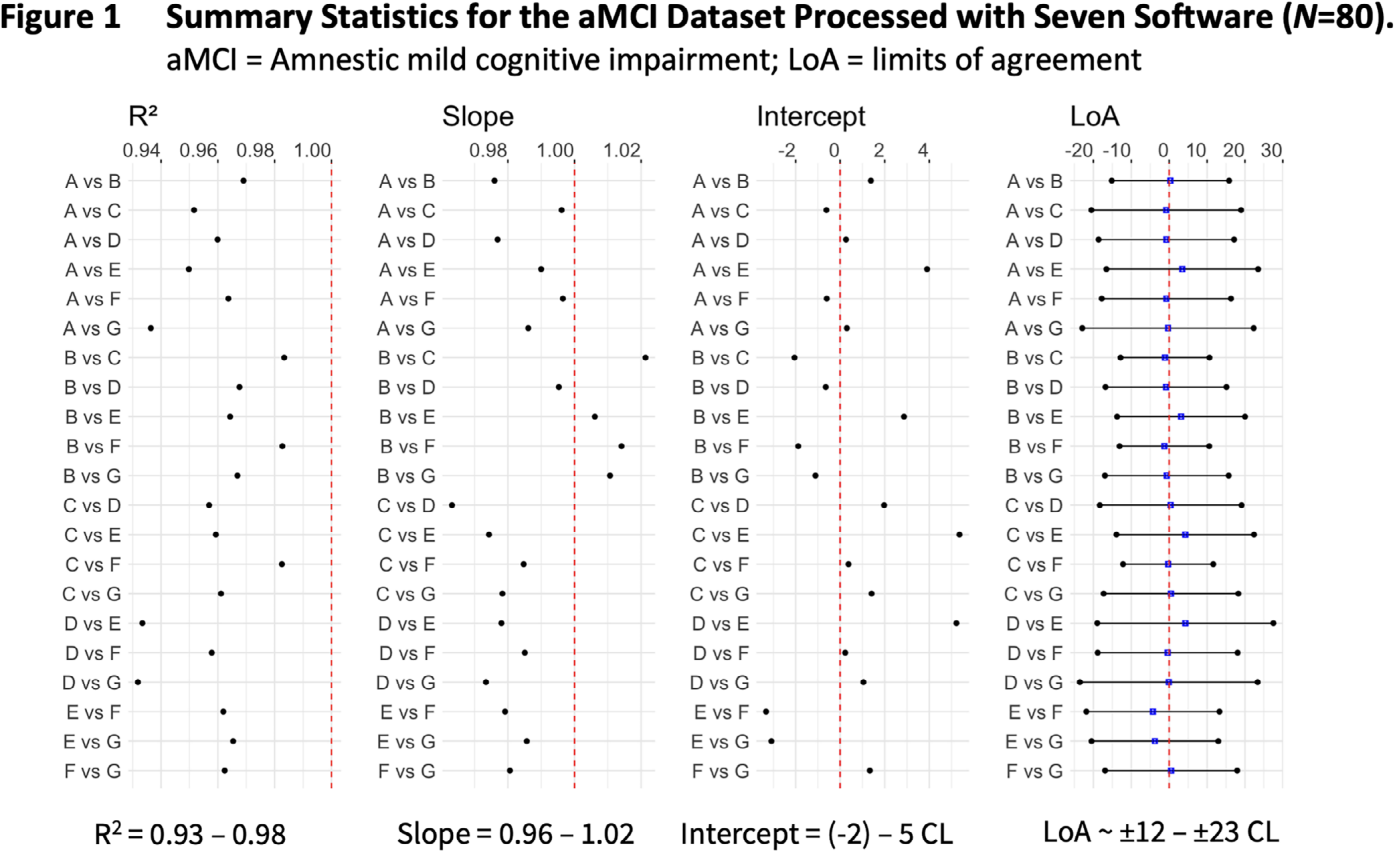# Interchangeability of Centiloid values from different research and commercial software using [^18^F]flutemetamol PET images

**DOI:** 10.1002/alz70862_110090

**Published:** 2025-12-23

**Authors:** Ariane Bollack, Christopher Buckley, Mark R Battle, Adam J. Schwarz, Oskar Hansson, Pierrick Bourgeat, Vincent Dore, Jurgen Fripp, Rachid Fahmi, Elena M Bonke, Lennart Thurfjell, William Balhorn, Mike Haas, Christopher Page, Gill Farrar

**Affiliations:** ^1^ GE HealthCare, Chalfont St Giles, Buckinghamshire UK; ^2^ Clinical Memory Research Unit, Department of Clinical Sciences Malmö, Lund University, Lund Sweden; ^3^ The Australian e‐Health Research Centre, CSIRO, Brisbane, QLD Australia; ^4^ The Australian e‐Health Research Centre, Commonwealth Scientific and Industrial Research Organisation, Brisbane, QLD Australia; ^5^ Siemens Medical Solutions USA, Inc., Molecular Imaging, Knoxville, TN USA; ^6^ Combinostics Ltd, Tampere, Tampere Finland; ^7^ Combinostics, Tampere, Tampere Finland; ^8^ MIM Software Inc., Cleveland, OH USA

## Abstract

**Background:**

Amyloid PET quantification using the Centiloid (CL) metric is becoming more prevalent and may have a role in clinical decision‐making. But what is the intrinsic confidence interval around a given CL value? To what extent is a CL value from one pipeline equivalent to that from another? We systematically assessed repeatability, reproducibility, and reliability across seven CL quantification pipelines applied to 210 [^18^F]flutemetamol scans.

**Method:**

Three datasets were used: (1) an AD test‐retest cohort (*N* = 10x2); (2) an amnestic MCI cohort (*N* = 80); (3) cases from the BioFINDER‐1 cohort enriched for amyloid loads around published positivity thresholds (0‐50CL) (*N* = 110). Three regulatory‐approved software (cPET, MIMneuro, *syngo*. MI Neurology) and four research pipelines (CapAIBL, rPOP, Amypype, SPM8) were evaluated. Dataset 1 was used to assess within‐subject repeatability. Dataset 2 was used to assess reproducibility in terms of absolute agreement in continuous CL values. Datasets 2 and 3 were used to assess reliability for dichotomizing amyloid scans into positive and negative. The latter was performed for three thresholds of 11, 25 and 37 CL.

**Result:**

The test‐retest analysis revealed absolute biases <5 CL, within‐subject coefficients of variation 2.6‐4.4% and 95% repeatability coefficients 8.1‐16.1 CL (Table 1). Strong group‐level associations in continuous CL estimates were observed across pairs of software (R^2^≥0.93, Figure 1), but 95% limits of agreement (LoAs) ranged between 12 and 23 CL. Agreement in +/‐ status between software was 92‐99% (kappa 0.84‐0.97) in dataset 2 and 75‐97% (kappa 0.48‐0.93) in dataset 3.

**Conclusion:**

CL values from most software pairs were interchangeable within the range of test‐retest variations, with high overall group‐level agreement. Ideally, all inter‐pipeline LoAs should be reduced to a similar level as test‐retest. Inter‐software reliability in dichotomization was >92% for scans across a wide range of CL values, but the percentage was lower for scans with amyloid loads closer to the range of typical positivity thresholds. Uncertainty estimates should always be considered when interpreting results.